# Revisiting the revisit: added evidence for a social chemosignal in human emotional tears

**DOI:** 10.1080/02699931.2016.1177488

**Published:** 2016-05-19

**Authors:** Noam Sobel

**Affiliations:** ^a^ Department of Neurobiology, Weizmann Institute of Science, Rehovot, Israel

**Keywords:** Social chemosignals, emotional tears, lacrimal secretions

## Abstract

In a study by Gelstein et al., we found that human emotional tears act as a social chemosignal. In the first of three different experiments in that study we observed that sniffing women’s emotional tears reduced the sexual attractiveness attributed by men to pictures of women’s faces. In a study partly titled “Chemosignaling effects of human tears revisited”, Gračanin et al. claim failed replication of this effect in a series of experiments, one they described as “exactly the same procedure” as Gelstein. Given that Gračanin et al. refused our extended offer to jointly replicate the experiment at our expense, we can merely comment on their effort. We find that Gračanin, who are not a chemosignaling laboratory, used methodology that falls short of standards typically applied in chemosignaling research. Thus, their experiments were profoundly different from Gelstein. Finally, we found that in reanalysing their raw data we could in fact replicate the effect from Gelstein. Thus, we conclude that the failed replication in Gračanin is neither a replication nor failed.

In a study by Gelstein et al. (2011) we reported on three experiments together suggesting that human emotional tears contain a social chemosignal. In Experiment 1 we observed a modest but significant effect whereby sniffing women’s emotional tears reduced the sexual attractiveness attributed by men to pictures of women’s faces (mean visual analogue scale (VAS) tears = 439 ± 118, mean VAS saline = 463 ± 125, *t*(23) = 2.5, *p* < .02, Cohen’s *d′* effect size = 0.51) (Figure 1(e)). In Experiment 2 we observed a slightly stronger effect whereby sniffing emotional tears altered psychophysiology and reduced levels of salivary testosterone in the context of emotional films (*d*′ = 0.55) (Figure 1(c)). In a third experiment we again observed a stronger effect whereby sniffing emotional tears altered patterns of brain activity while observing sad faces, as measured with fMRI (*d*′ = 0.79). An independent group recently published a successful replication of the effect on testosterone, whereby merely sniffing women’s emotional tears again significantly reduced levels of free testosterone in men (Oh, Kim, Park, & Cho, 2012) (Figure 1(d)). Given that men report stronger attraction to women’s faces when their testosterone levels are high (Welling et al., 2008), this replication also indirectly supports Gelstein Experiment 1. Finally, an additional group reported an effect in mice similar to the effect we first found in humans. Specifically, sniffing a peptide secreted from the lacrimal gland and released into tears of juvenile mice exerted a powerful inhibitory effect on adult male mating behaviour (Ferrero et al., 2013) (Figure 1(b)). A functional chemosignaling role for rodent tears had previous support (Kimoto, Haga, Sato, & Touhara, 2005; Shanas & Terkel, 1997; Thompson, Napier, & Wekesa, 2007) (Figure 1(a)), and now it was significantly bolstered. This recent discovery was particularly gratifying for us because our stated conclusion in Gelstein was that the very basic biological function we identified for emotional tears implies that emotional tears are not necessarily a uniquely human phenomenon. We think that tears evolved as a sort of “chemical blanket” protecting the animal against aggression (sexual and other), and this biological driving force remains common across rodents and humans. This notion of tears as a social chemosignaling mechanism common across mammalian species is in contrast to the view of emotional tears as a uniquely human phenomenon, as echoed in the book title “Why only humans weep” (Vingerhoets, 2013) by Dr Ad Vingerhoets. In Gračanin et al., the group of Dr Vingerhoets set out to recreate our Experiment 1 from Gelstein, and stated that despite applying methods “completely the same” as in Gelstein, they failed to observe an effect. After obtaining the Gračanin raw data, here we argue that although Gračanin did not replicate Gelstein methods, they in fact did support the Gelstein result.

**Figure 1.**
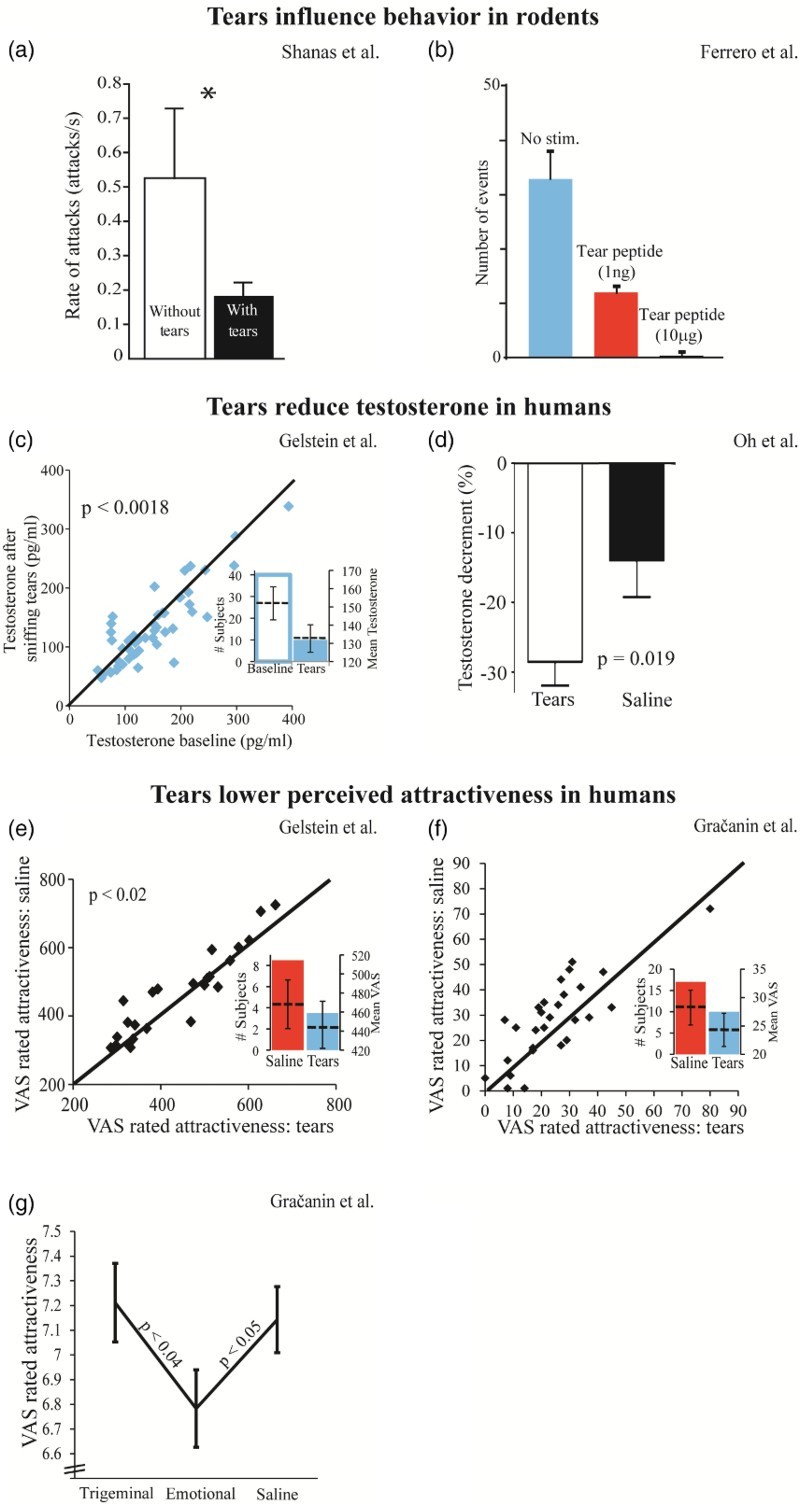
Chemosignaling effects of tears replicate across species and studies. (a) Mole-rats that cover themselves with their own tears are attacked less frequently by dominant males. Adapted from (Shanas & Terkel, 1997). (b) Replication across species: Mouse pups covered with a peptide in tears are subject to less sexual behaviour by adult males. Adapted from (Ferrero et al., 2013). (c) Sniffing emotional tears obtained from women reduced free testosterone in men. Adapted from (Gelstein et al., 2011). (d) A replication within species: Sniffing emotional tears obtained from women reduced free testosterone in men. Adapted from (Oh et al., 2012). (e) Sniffing women’s emotional tears reduced the sexual attractiveness attributed by men to pictures of sad women’s faces. Adapted from (Gelstein et al., 2011). (f) A replication within species: Sniffing women’s emotional tears reduced the sexual attractiveness attributed by men to a picture of a woman. Adapted from Gračanin et al. Experiment 3, ratings of picture #14. (g) A replication within species: Sniffing women’s emotional tears reduced the sexual attractiveness attributed by men to pictures of women. Trigeminal tears failed to induce the same effect. Adapted from Gračanin et al. Experiment 2, Sample 1.

## 1. Gračanin Experiment 3 differed from Gelstein in a major methodological aspect, and once accounted for, Gračanin supported Gelstein

Gračanin highlight their Experiment 3 as the one experiment in their study that was “completely the same” as Gelstein. In fact, it was rather different. First, it was conducted under an entirely different context. Context is a major shaping factor in sensory perception in general, and in chemosensation in particular (de Araujo, Rolls, Velazco, Margot, & Cayeux, 2005). Moreover, many human social chemosignaling effects materialize only in specific contexts but not others (Bensafi, Brown, Khan, Levenson, & Sobel, 2004; Jacob, Hayreh, & McClintock, 2001; Lundstrom & Olsson, 2005; Olsson, Lundström, Diamantopoulou, & Esteves, 2006; Saxton, Lyndon, Little, & Roberts, 2008). With this fundamental notion of context-dependence in mind, all our experiments with tears as a potential chemosignal were in the context of sadness, as this was the ecologically valid context of the tears we harvested. On each trial of the Gelstein face-rating experiment participants viewed utterly sad faces (morphed with neutral images) and rated them for sadness and subsequently viewed neutral faces and rated them for attraction (interleaved counterbalanced). Thus, sadness was always on the mind of participants and served as a dominant contextual framework of the experiment. The experiments reported by Gračanin never introduced sad context. They did not show sad faces, and did not obtain sadness ratings. Thus, participants of Gelstein and Gračanin were in a different mindset, and these two experiments were not “completely the same”.

The difference between these two studies, however, extended far beyond context to include major methodological aspects, and key amongst them was a profound difference in stimuli: Whereas Gelstein used a publically available set of face images (the NimStim set (Tottenham et al., 2009)), Gračanin presented their subjects with a different novel set of face images.

So the Gelstein and Gračanin experiments were methodologically different (different context and questions: no “rate sadness”, different stimuli: 18 novel faces, and additional methodological differences described later), but do these differences matter? It is difficult to compare the face ratings provided in Gelstein and Gračanin because they used different rating methods and scales (Gelstein used a 14 cm long VAS without digits or markers, then converted into milimetric values. Gračanin used 10-point and 100-point scales). To address this, we first combined the 18 faces used by Gračanin Experiment 3 with the 18 faces used in Gelstein, and asked 15 male subjects to rate sexual attractiveness of the 36 faces on a scale from 1 (not at all) to 100 (very high). This provides for a uniform assessment of the stimuli used across studies. We observed an overwhelming difference across stimuli sets. The faces used by Gračanin were rated double sexually attractive compared to the faces used by Gelstein (mean attractiveness Gračanin = 60.66 ± 10.46, mean attractiveness Gelstein = 32.89, *t*(14) = 9.4, *p* < .0001). To address the possibility that this difference reflected a cultural difference between Holland and Israel we compared the ratings of the Gračanin faces by Dutch and Israeli men. We found that these groups use scales differently (mean Dutch rating = 41.64 ± 15.47, mean Israeli rating = 60.66 ± 10.64, *t*(34) = 4.3, *p* < .0001) (thus justifying the unified test we conducted), but that they are highly correlated in their rankings (Spearman Rho = 0.87, *Z* = 3.6, *p* < .003). In other words, Dutch and Israeli men do not rank-order women’s attractiveness differently. Taken together, these results introduce the experimental hazard of off-scale stimuli in Gračanin. We next asked whether any of the faces used by Gračanin falls within the distribution of the Gelstein faces (within one standard deviation of the mean), and identified one stimulus alone, Face #14 from the Gračanin experiment. This face obtained a mean attractiveness rating of 40.1, not significantly different from the mean of the Gelstein faces (one-sample sign, *p* = .3), and significantly different from the mean of the Gračanin faces (one-sample sign, *p* < .0001) (Supplementary File 1). We then asked whether emotional tears influenced the perception of this one face in the Gračanin results. Consistent with Gelstein, we observed that sniffing emotional tears significantly reduced the attractiveness attributed to this face in the Gračanin raw data (mean saline = 28.25 ± 16.2, mean tears = 24.32 ± 15.45, one-tailed paired *t*-test; *t*(27) = 2.05, *p* = .025, Cohen’s *d*′ = 0.393) (Figure 1(f)) (given that this is an intended replication, we are obliged to apply one-tailed tests, that said, note that this survives a two-tailed statistic as well). In other words, when the off-scale stimuli effect introduced by Gračanin was accounted for, the Gračanin data supported Gelstein (Figure 1(f)).

## 2. Gračanin Experiment 2 combined two experiments in a statistically forbidden manner. Once separated, Gračanin again supported Gelstein

Getting a sense of the data in Gračanin is not completely intuitive because their manuscript is without figures, so the distribution and variance of the data were not immediately apparent. With this in mind, something in Gračanin Table 3 regarding their Experiment 2 was noticeable: Experiment 2 was in fact two separate experiments, referred to by Gračanin as Sample 1 and Sample 2. Sample 1 and Sample 2 were conducted 18 months apart, with different non-overlapping tear donors, with some overlapping but some different questions, and with a different statistical design. Gračanin Table 3 indeed separately reports Sample 1 and Sample 2 results for one question that overlapped across “Samples” (“Punishment”), but on the question of replication, namely “rate attractiveness” that also overlapped across “Samples”, they combine Sample 1 and Sample 2 results. To first ask whether these data sets can be combined as done by Gračanin, we used an Equality of Variance *F*-test applied to the attractiveness ratings obtained following emotional tears or saline in Sample 1 and Sample 2. This uncovered a profound large difference in variance across these experiments (Sample 1 Variance = 55.36, Sample 2 Variance = 139.79, *F*(48) = .4, *p* = .0017). In other words, combining the results of Sample 1 and Sample 2 as done by Gračanin was statistically forbidden (Supplementary File 2 extends this analysis). We then continued to examine each of these experiments alone. An analysis of Sample 1 alone successfully replicated the effect reported in Gelstein (mean attractiveness tears = 6.78 ± 0.78, mean attractiveness saline = 7.14 ± 0.66, one-tailed *t*-test: *t*(47) = 1.73, *p* = .045) (Figure 1(g)), yet Sample 2 did not (mean tears = 75.29 ± 11.29, mean saline = 76.53 ± 12.57, one-tailed *t*-test: *t* = .365, *p* = .358). As expected from the above Equality of Variance *F*-test, the standard deviations in Sample 2 were about double those in Sample 1, and indeed combining Samples 1 and 2 obscured the effect from Sample 1 alone (of course, all this after multiplying Sample 1 ratings by 10 to equate scales (1–10 vs. 1–100). Without this step the differences are even bigger). We repeated this analysis using the novel composite sexual measure used in Gračanin but not Gelstein, and obtained even slightly stronger results (mean tears = 67.09 ± 12.46, mean saline = 73.14 ± 11.32, one-tailed *t*-test: *t*(47) = 1.78, *p* = .041). To reiterate, despite the significantly reduced power of the across-subjects design used by Gračanin compared to the within-subjects design in Gelstein (see comment on power in Supplementary File 3), and further despite the chemosignaling methodological flaws in Gračanin as discussed next, the effect nevertheless immerged (Supplementary File 4 contains all steps from raw data file to replication). Sniffing emotional tears compared to saline reduced the attractiveness attributed to images in Gračanin Experiment 2, Sample 1 (Figure 1(g)). Finally, to ask if we can also learn something new from the study by Gračanin, we added into the analysis the novel condition of trigeminal tears tested by Gračanin but not Gelstein. Whereas the interaction statistic was not significant (*F*(2, 71) = 2.13, *p* = .12), planned comparisons revealed that trigeminal tears were not different from saline (mean trigeminal tears = 7.21 ± 0.79, mean saline = 7.14 ± 0.66, one-tailed *t*-test: *t*(47) = .33, *p* = 0.37), but significantly different from emotional tears (mean tears = 6.78 ± 0.78, mean trigeminal tears = 7.21 ± 0.79, one-tailed *t*-test: *t*(48) = 1.92, *p* = .03) (Figure 1(g)). In other words, this study added an important control that further strengthens the notion of a chemosignal in human emotional tears, and suggests absence of this signal in trigeminal tears (Figure 1(g)).

## 3. Gračanin falls short of common methodological standards in the study of human social chemosignaling

As we have detailed so far, once the methodological difference in stimuli was accounted for, Gračanin supported Gelstein in Experiment 3. Moreover, once Experiment 2, Sample 1, and Sample 2 were disentangled, Gračanin supported Gelstein in Experiment 2. These effects materialized despite several methodological differences across studies. These differences mostly reflect that Gračanin are not a lab that studies chemosignaling. For example:

1. When chemosignal-induced arousal or its ensuing rated attraction are the measures of interest, it is absolutely critical to test subjects at the same time of day across days and conditions (tears and saline), as was done in Gelstein. This is because men’s testosterone fluctuates on a circadian cycle, with a morning peak and an evening lull (Cooke, McIntosh, & McIntosh, 1993). As noted, men report stronger attraction to women’s faces when their testosterone levels are high (Welling et al., 2008). Thus, if men are tested with tears in the morning and saline in the evening, this confound is sure to eradicate the Gelstein effect. In addition to testing at the same time of day, it is important to test at the nearest possible calendar dates across conditions, ideally day-after-day (as in Gelstein). This is because although testosterone is relatively stable over time, its stability is still higher across days than across weeks (Dabbs, 1990). Gračanin did not maintain day-after-day testing, mostly testing one week apart. But did this actually hamper the experiment by adding variance? Here we can directly test this: Regardless of an effect, the correlation between test #1 and test #2 in Gelstein was *r* = .93 yet the correlation between test #1 and test #2 in Gračanin was *r* = .79, and these correlations are significantly different (*Z* = 1.98, *p* < .05). In other words, the Gračanin inappropriate design added a profound extent of unwanted variance that likely obscured effects.
2. Objective evidence that subjects in Gračanin actually sniffed the stimuli is lacking. In Gelstein, subjects took 10 fixed-duration tone-guided sniffs of the stimulus with ∼40 seconds between sniffs, and critically, after every sniff rated the stimulus for intensity, pleasantness, and familiarity. These ratings serve two purposes: First, they force subjects to attend to sniffing and odour content, thus assuring effective and consistent exposure. Second, they allow us to compare the ratings attributed to the stimulus (tears) and control (saline) in order to assure no contamination or perceptual differences (results of these comparisons all presented in Gelstein). Finally, in all studies we also measured concurrent nasal airflow using spirometry in order to assure equal sniffing across conditions. Gračanin et al. did next to none of this. Their subjects were instructed to take only three sniffs in Experiment 1 and Experiment 2, Sample 2 (10 in the others), they say nothing of an inter-sniff-interval that is critical to prevent habituation, they provided no ratings (minimizing attention and preventing tests of contamination), and did not measure airflow. This constitutes a significant added source of potential variance in Gračanin.
3. Lastly is the issue of contamination. Social chemosignals are supremely potent (Laska, Wieser, & Salazar, 2005) with evidence from insects implying detectability of a single molecule (Kaissling & Priesner, 1970). Chemosignaling labs are all too familiar with this experimental hazard, and conduct themselves accordingly. We treat chemosignals as wet labs treat radioactive material, and relevant procedures were detailed throughout Gelstein et al. Given that Gračanin are not a chemosignaling lab, it is unsurprising to see no treatment of these issues in their manuscript (Table 1).

**Table 1.** Revisiting the revisit: Profound methodological differences between Gračanin et al. and Gelstein et al.

Method	Gračanin Experiment 1	Gračanin Experiment 2	Gračanin Experiment 3	Gelstein
Within-subjects design	✘	✘	✓	✓
Subjects view sad faces	✘	✘	✘	✓
Subjects view morphed faces	✘	✘	✘	✓
Subjects view half-naked women	✓	✓	✘	✘
Subjects rate sadness	✘	✘	✘	✓
Subjects tested at exact same time of day, day-after-day, across conditions and days	✘	✘	✘	✓
Subjects rate stimuli for pleasantness/intensity/familiarity to assure exposure	✘	✘	✘	✓
Precautions to prevent contamination	**?**	**?**	**?**	✓

Note: Table 1 lists some key differences across experiments. Note that this table is not exhaustive, as it does not detail several steps conducted in Gračanin but not in Gelstein, such as an entire added condition of reflexive tears. This and other additions are both interesting and important in themselves, but they too take away from the element of exact replication and instead constitute a different study all together. Viewing Table 1 is at odds with the Gračanin et al. statement regarding methods that were “completely the same” as Gelstein et al.

## Discussion

We have detailed how Gračanin was profoundly different from Gelstein, and fell short of methodological standards typically applied in social chemosignaling research. Nevertheless, when we corrected for the off-scale stimuli effect introduced by the face stimuli in Experiment 3, we found that Gračanin supported Gelstein (Figure 1(f)). Similarly, when we corrected for the averaging in Experiment 2, we again found that Gračanin supported Gelstein (Figure 1(g)). Note, the power of these “replications” in Gračanin is not overwhelming, and we would indeed not publish or recommend publishing a manuscript claiming a chemosignal in emotional tears based on these results alone. In turn, these effects are meaningful enough so as to have prevented a claim of failed replication in our opinion.

The issue of replication has commanded massive recent attention (Anderson et al., 2016; Button et al., 2013; Gilbert, King, Pettigrew, & Wilson, 2016; Open Science, 2015), and we think this will do only good for science in the long run. In our lab we have indeed increased the extent of internal replications we now perform before publication (this occurred well before Gračanin), and this is likely a good thing. That said, we must take caution not to throw the baby out with the bath water. For example, if Gračanin were to try and also replicate the recent demonstration of a chemosignaling sexual inhibitor in mouse tears (Ferrero et al., 2013) (Figure 1(b)), they would likely fail that replication as well. This, however, would not reflect on the validity of Ferrero et al., but rather reflect that Gračanin are not a rodent lab with molecular tools. Similarly, Gračanin are not a social chemosignaling laboratory, and this was very evident in their methodology. Does this imply that they cannot under any circumstances try to replicate our work? No, it does not. Such replication efforts, however, should follow certain standards (Kahneman, 2014). One option would be for Gračanin to send a student to our lab in order to try and replicate together. It so happens that we in fact extended such an offer to Gračanin, including the willingness to fully fund the endeavour, yet our offer was declined. As to the scientific question at the heart of this issue, it remains our view that human emotional tears function as a social chemosignal, a function common across mammalian species. We find that the raw data in Gračanin (Figure 1(f) and 1(g)) does not negate this hypothesis, and if anything, it supports it.

## Supplementary Material

PCEM_1177488_Revised_Supplementary_Material_12-4-16.zip
