# Supplementary material for: Revisiting the revisit: added evidence for a social chemosignal in human emotional tears
Source: Cogn Emot. 2016 May 19;31(1):151–7. doi: 10.1080/02699931.2016.1177488 (PMC5215200; doi:10.1080/02699931.2016.1177488)

Supplementary File 2 for "Revisiting the Revisit: Added Evidence for a Social Chemosignal in Human Emotional Tears"

**Experiment 2 Sample 1 and Sample 2 cannot be combined**

To compare Sample 1 and Sample 2 data we first multiplied the Sample 1 data by 10, as it is reported on a scale from 1-10, and Sample 2 on a scale from 0 to 100. We then submitted these data to an Equality of Variance F test, and this uncovered a profound large difference in variance across these experiments (Sample 1 Variance = 55.36, Sample 2 Variance = 139.79, F(48) = .4, p = 0.0017). Here we further investigate the differences between these distributions using a Kolmogorov-Smirnoff test. We compared all the ratings in each Sample, and rejected the hypothesis that the ratings (samples) come from the same distribution with p-value smaller than 0.001. We further tested the differences between the two conditions (tears / saline) in the two parts, and found that both the tears and the saline were not originated from the same distribution with p-values of 0.004 and 0.003 respectively. We concluded that the two parts of the experiment were essentially two different experiments.


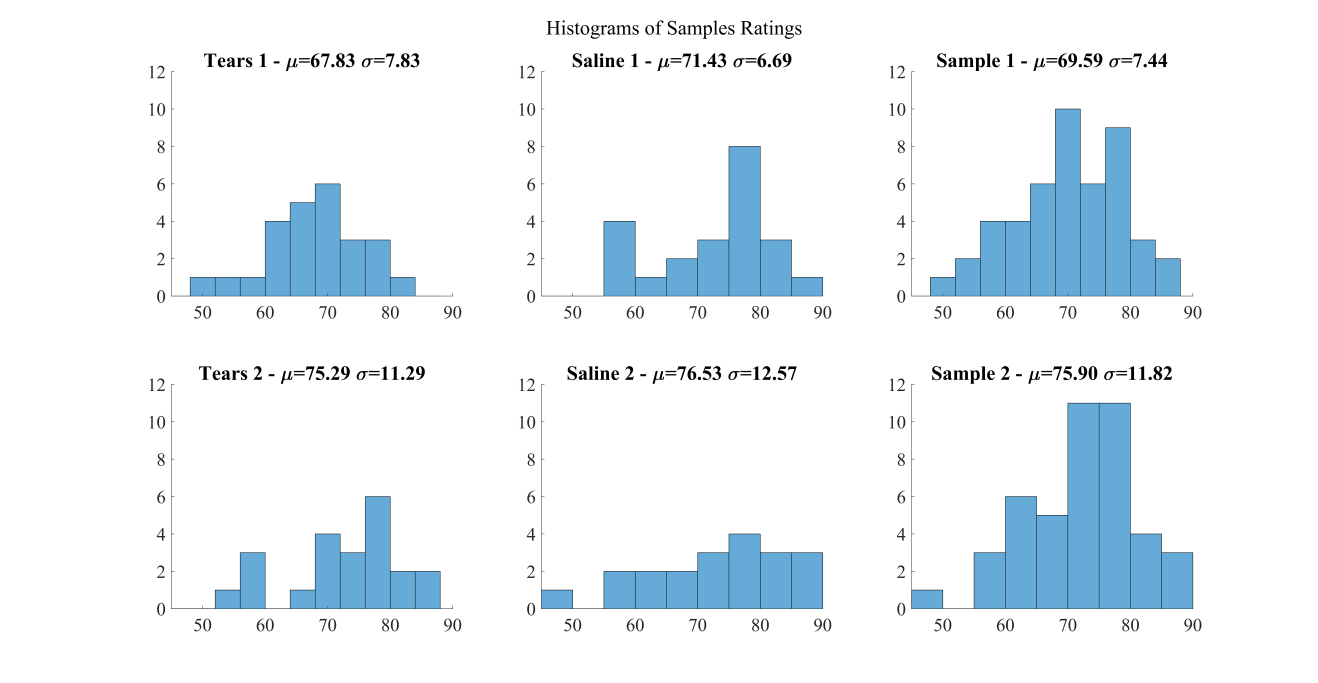


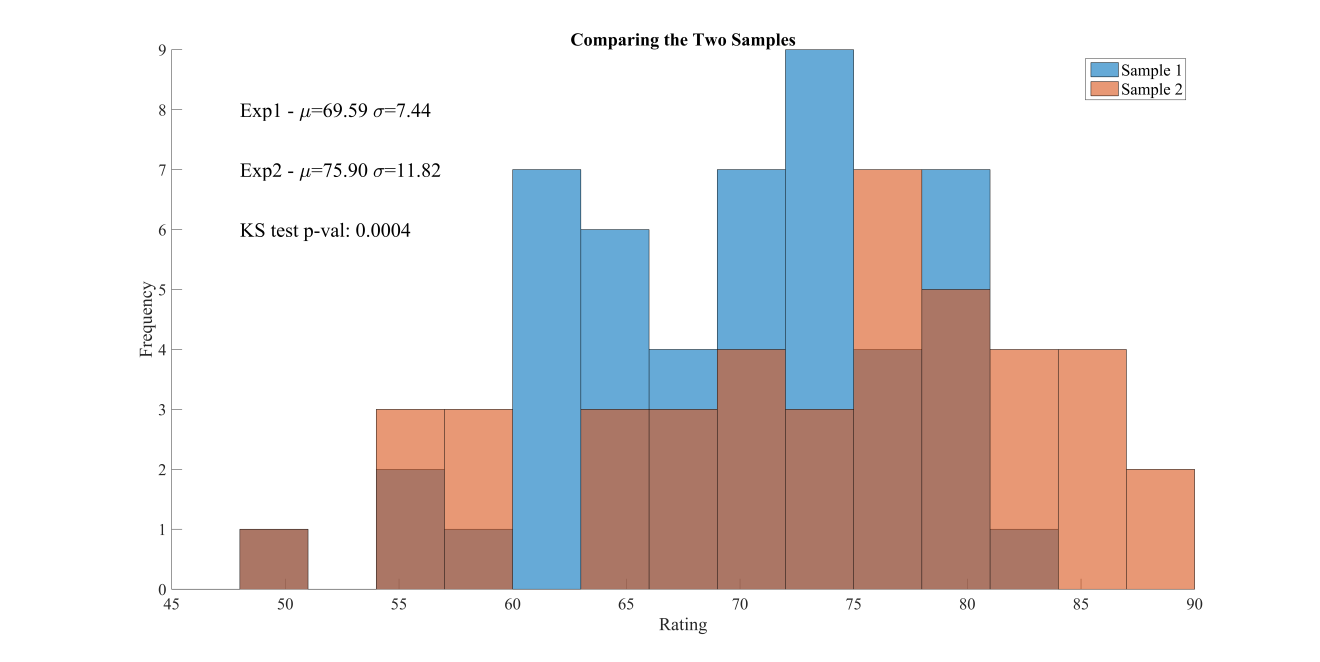

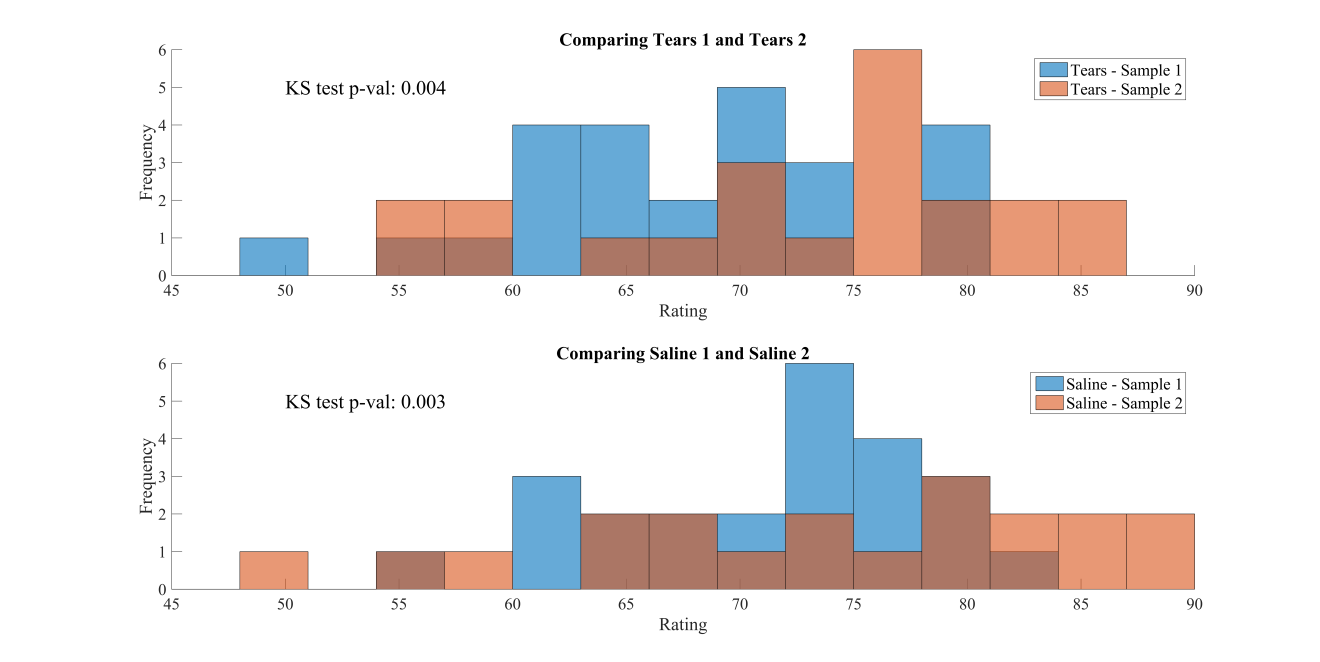

Supplement: PCEM_1177488_Revised_Supplementary_Material_12-4-16.zip [file pcem_a_1177488_sm7733.zip › SuppFile2PV.docx]
