# Supplementary material for: Revisiting the revisit: added evidence for a social chemosignal in human emotional tears
Source: Cogn Emot. 2016 May 19;31(1):151–7. doi: 10.1080/02699931.2016.1177488 (PMC5215200; doi:10.1080/02699931.2016.1177488)

Supplementary File 3 for "Revisiting the Revisit: Added Evidence for a Social Chemosignal in Human Emotional Tears"

**Power Analysis**

We would like to stress that statistical power is **NOT** a major problem with Gračanin et al. More specifically, we speculate that had they studied additional subjects to meet the power requirements, their Experiment 3 would still yield a null result, reflecting its various methodological shortcomings as detailed in the manuscript. Thus, we are not making a big thing out of the power issue. That said, if one sets out to conduct a replication, the first order of business is an a-priori power analysis, and not doing so was just one more instance where Gračanin et al. fell short of experimental standards. Moreover, there sample-sizes fell short of power analysis recommendations.

To conduct an a-priori power analysis for a replication of Gelstein et al., we used G-Power (http://www.gpower.hhu.de/en.html) according to:

Faul, F., Erdfelder, E., Lang, A.-G., & Buchner, A. (2007). G*Power 3: A flexible statistical power analysis program for the social, behavioral, and biomedical sciences. Behavior Research Methods, 39, 175-191.

For a replication using a within-subjects design at the power reported in Gelstein et al., we find that one should test 36 subjects for a one-tailed test, or 44 subjects for a two-tailed test:


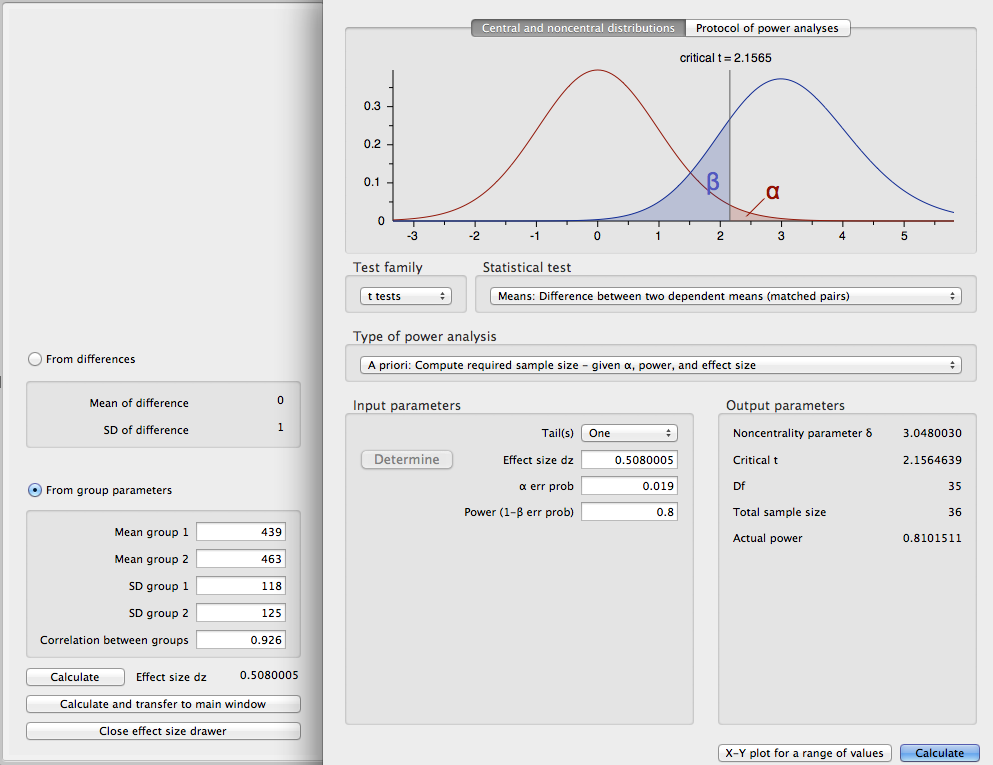


As to the odd methodological decision by Gračanin et al to test replication of our within-subjects design using an across-subjects design, one can't really say how to do an equivalent between subjects test unless you know how large the between subjects variance is relative to the within subject error variance. Our only poor estimate of this is if we take the first visit only from our 24 subjects, allowing for a 12-subject across-subjects test. We then aim for the same alpha as in the within subjects test. This analysis implies that Gračanin et al would need to test 228 individuals (114 in each group) in order to test our within-subjects result.


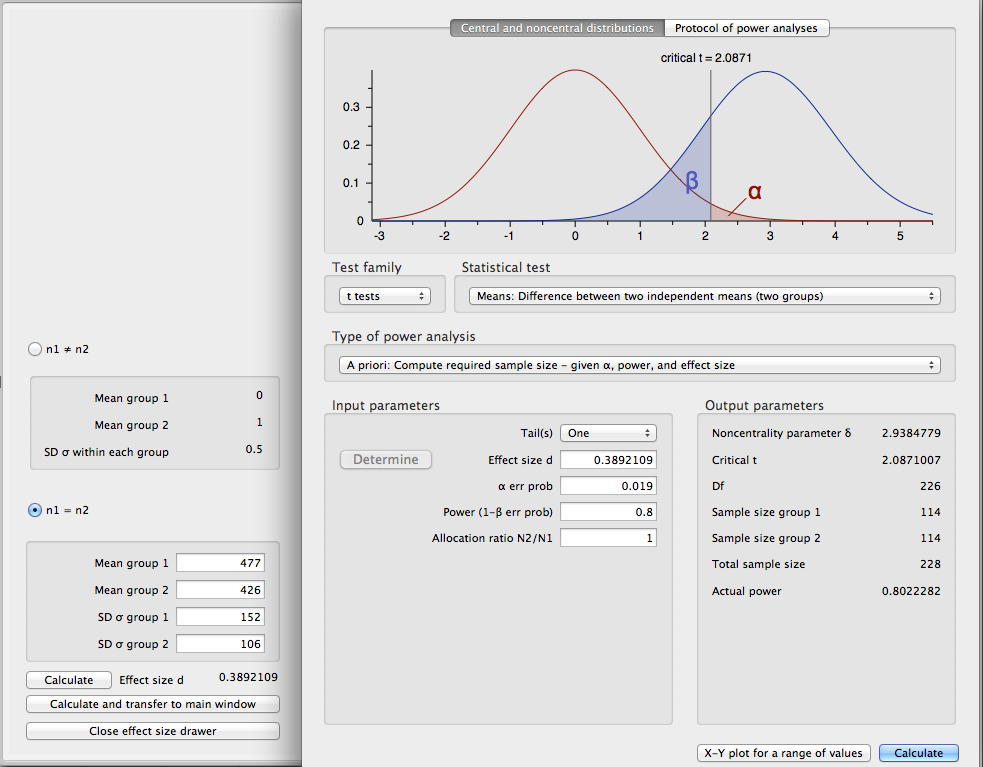

Supplement: PCEM_1177488_Revised_Supplementary_Material_12-4-16.zip [file pcem_a_1177488_sm7733.zip › SuppFile3PV.docx]
