# Supplementary figures and images for: Revisiting the revisit: added evidence for a social chemosignal in human emotional tears
Source: Cogn Emot. 2016 May 19;31(1):151–7. doi: 10.1080/02699931.2016.1177488 (PMC5215200; doi:10.1080/02699931.2016.1177488)

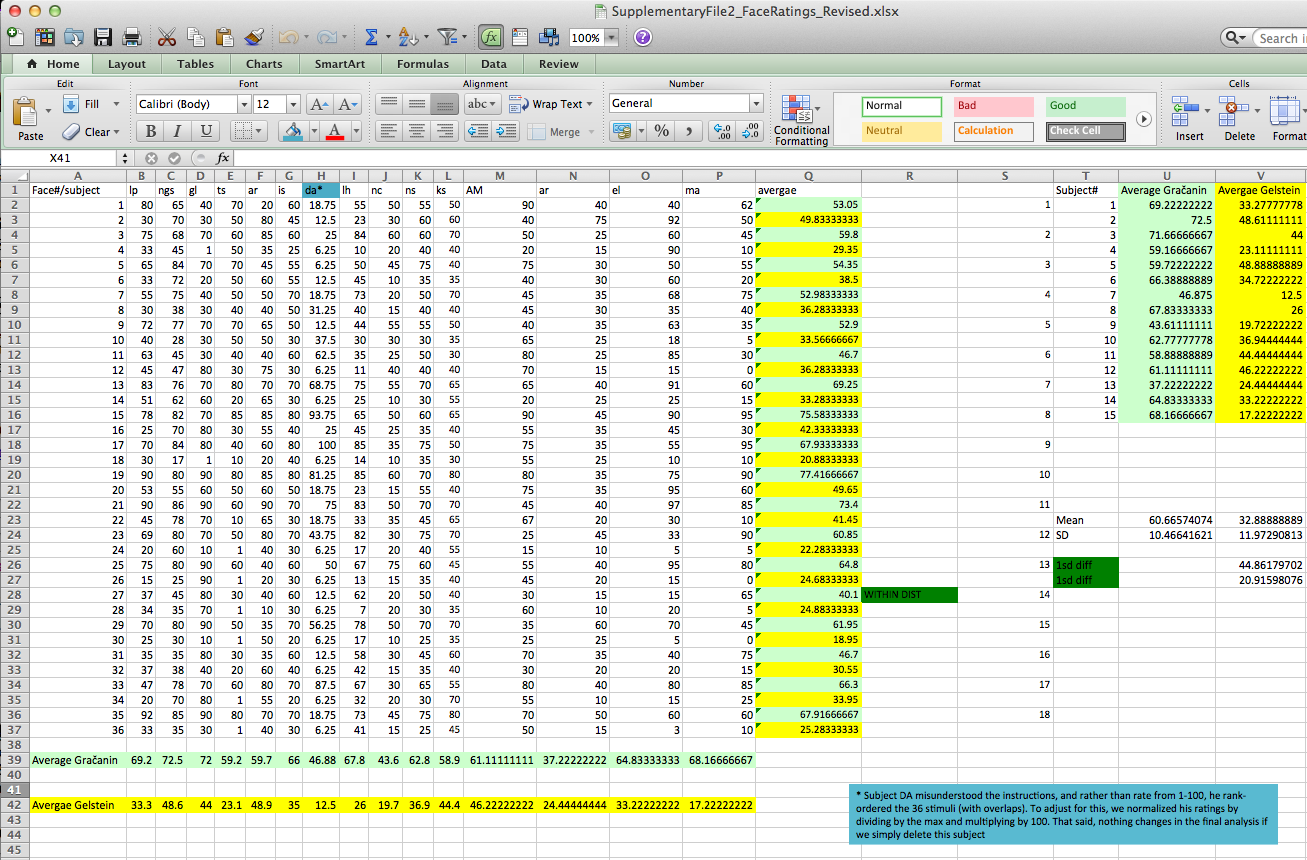

Supplement: PCEM_1177488_Revised_Supplementary_Material_12-4-16.zip [file pcem_a_1177488_sm7733.zip › SuppFile1PV.png]

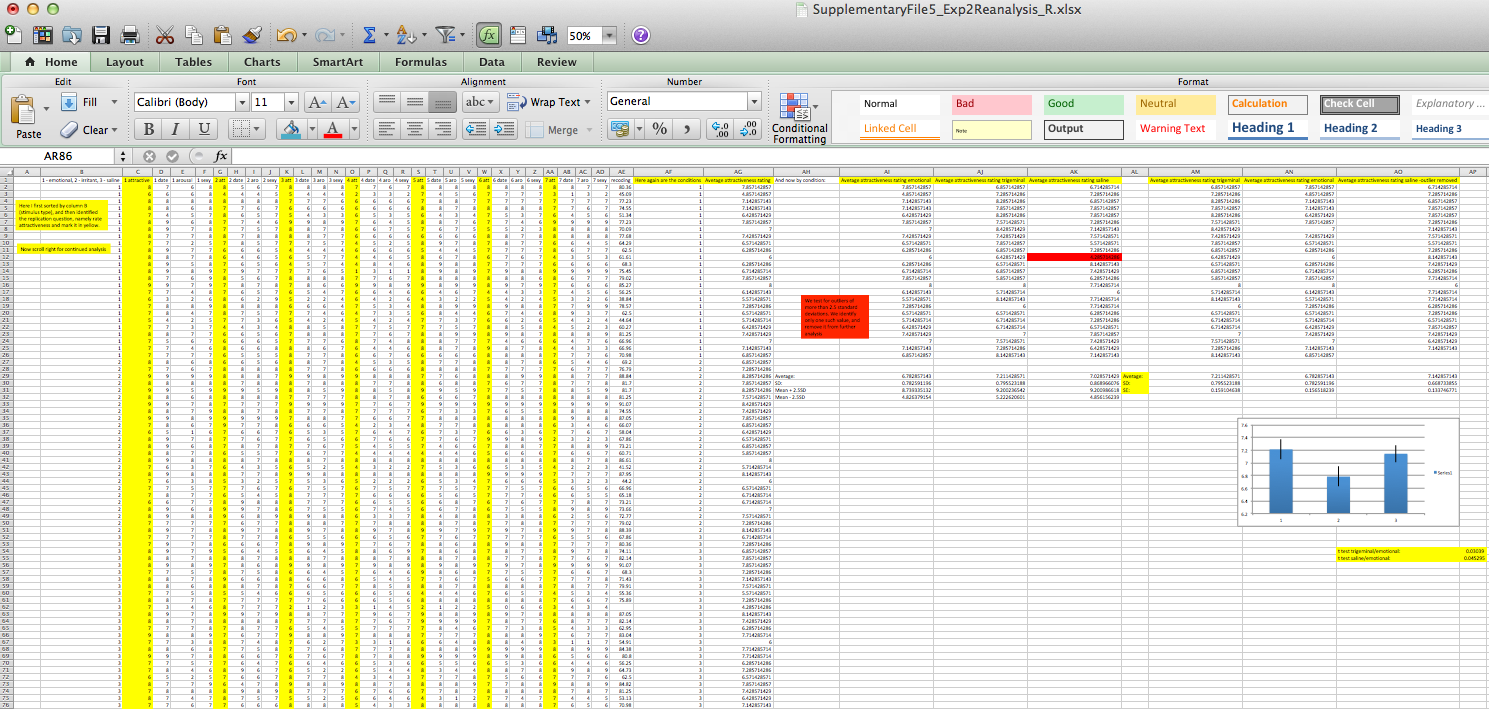

Supplement: PCEM_1177488_Revised_Supplementary_Material_12-4-16.zip [file pcem_a_1177488_sm7733.zip › SuppFile4PV.png]
